# Case of Intermittent Fever, Accompanied with a Sympathy of Organs Which Have No Sensible Connection with Each Other

**Published:** 1809-12

**Authors:** M. Cazals

**Affiliations:** Physician at Agde


### *Case of Intermittent Fever, accompanied with a Sympathy of Organs which have no sensible Connection with each other. By* M. Cazals, *Physician at Agde.*

( From the Journal de Medecine. )

PERNICIOUS intermittent fevers, descriptions of which have been faithfully recorded by medical practitioners of the first eminence, assume the characters of every disease, are concealed under different forms, and would end in carrying off the patient, if their anomalies were not conquered by the treatment dictated by the nature of the fever to be combated.

Diseases of this kind form an order by themselves, and which Mercatus, Heredia, Morton, Werlhoff, Medicus, Lancisi, Porti, Senac, Lauther, Cleghorn, Barthez, Dumas, Baumes, Alibert, Doublé, &c. have enriched with various important facts, besides pointing out some varieties not hitherto observed.

It occurs to me, that there is still a desideratum to this order; order; and I hope I shall be forgiven in endeavouring to supply it by the following case, which occurred under my own observation.

M. François Valat, a farmer at Agde, aged 76, below the middle stature, habitually subject to ring-worms, (but which did not give him much inconvenience) of a delicate constitution, but not sickly, was seized in 1806 with an intermittent fever of the pediculosus kind, accompanied by sympathetic pains, and pruritus round the neck. Shame prevented him from mentioning his disease for some time; but worn out at last by the pain he endured, I was sent for. Six hours elapsed before I could see him; he was then in a violent agitation, complaining of burning heat, and exhibited a pruriginous eruption on the neck and shoulders. The itching was so intense that he could not refrain from scratching a single moment.

Every pimple that he raised, produced a swarm of vermin, which were multiplied with astonishing rapidity. The number of lice to which the body of this unfortunate person gave birth was incredible; the hollows around his eyes swarmed with them.

My appearance excited so lively an emotion in the patient, that when I approached the bed he trembled and shivered with as much violence as on the first appearance of the fever; his speech was broken, and it was some time before he could articulate sufficiently to tell me his case. I examined his body, and saw such a quantity of vermin on his neck and shoulders, that I was petrified with horror. Thousands of them came from under the skin, in proportion as he scratched himself; they were large and white, and seemed to be a distinct species from those found in the head.

But the most singular circumstance of all was, that the patient never scratched himself, without causing an acute pain in the great toe of the right foot, which ascended upwards by the right side of the pelvis, and affected the stomach to such a degree, that he could not swallow the smallest quantity of liquid without danger of suffocation. The instant that a drop of moisture touched the cardiac orifice, he exclaimed that the attendants were squeezing his great toe, where he felt the most acute pain; and when it was actually squeezed with some force, he swallowed with more ease.* We

* The stomach, of all the viscera, is that in which we see most frequently certain sympathetic communications with organs that have no sensible connection with it. Barthez, Nouveaux Elemens de la Science de l'Homme.

We may refer this part, which has certainly some analogies in various collections of cases, to the correspondence of the different affections of two organs not sympathetic, which habit establishes as an effect of repeated successions of these affections, and to the idiosyncracy or individual constitution of those in whom it is observed.

The following day there was an entire cessation of fever and of pain; no increase of pustules nor of lice.

Next day all the former symptoms returned; he evinced the same restlessness, and every thing fretted him: intense heat with high fever; acute pain in the great toe of the foot; pimply eruption, and vermin. All these concomitants attended and ceased with the fever. Symptoms so varied, and so dissimilar, did not keep me long in uncertainty as to the diagnosis and treatment. In any other case I could not have known whether to regard the pain in the great toe as rheumatism, and to treat it as such, or as a mere sympathetic affection. In the present case I adopted the latter opinion; it was necessary, therefore, to avail myself of the apyrexia, which was marked by a great feebleness in the lower extremities, by slight shiverings, and by transient heats in the palm of the hands and soles of the feet.

There came on, from time to time, nausea, with a discharge of mucous matter. I prescribed the methods pointed out in similar circumstances; ipecacuanha, joined to antimonial tartrite of potash.

The patient having great aversion to vomiting, could not be prevailed on to take this medicine. I did not hesitate to point out to him his danger; I beseeched him to give up an opinion which could not but prove fatal to him. All was in vain, as he persisted in his resolution.

Quinquina was suggested, but with no avail; at last, however, I succeeded in overcoming his repugnance.

In the apyrexia, the quinquina was administered under every form; in decoction externally, to kill the vermin; and internally in substance. Under this form, the febrifuge was raised to the dose of ten drachms in twenty-four hours. The fever, the lice, and the pains were removed. In order to avoid a relapse, I advised my patient to use the specific on certain days; for once he adhered to my prescriptions. From this moment he regained his former lusty appearance.

From this case, may we not give to the fever the name of *phthiriasis,* and use quinquina against the morbus pediculosus, which perhaps would act better against this affection than the remedies hitherto employed?

Lice are multiplied in the manner of ovipari: they lay their eggs on the hair or the skin, in the matter of perspiration, or on the clothes. The heat of the body hatches them, and every egg produces a fresh animal. Want of cleanliness favours their propagation; habit of the body may also contribute to it, a habit, the precise nature of which cannot be determined, but which observation puts beyond a doubt. This disease attacks irritable temperaments, rarely old men; and among the latter, only those who have something cachectic in their constitution. This was not the case with my present patient; the only thing that could be imputed to him were ring-worms. Was this cause sufficient? Several practitioners of eminence assert, that those who are affected in this manner, more easily contract the morbus pediculosus; it remains for observation and experience to give us further information.

I shall not detain my readers with other examples of sympathies between various and remote organs; these cases are rare and singular; I shall call their attention, however, to the case of a man mentioned by Hales, who felt an acute pain at the top of the left shoulder when he scratched a pimple which was a litle below the exterior side of the right knee, and to the fact stated by Cullen, namely, that if a dog licked his hand gently, he felt a tickling in the sole of his feet.

A great uumber of singular diseases make us acquainted, by means of symptoms, independent of the generic forms essentially characteristic of these diseases, with true sympathies between organs, which in other respects have no sensible relation.

Abscesses, succeeding to wounds of the head, are symptoms of this nature. It is impossible to explain, or rather to refer this phenomenon to any known genera of sympathies of organs. Nothing here advanced, can give us any assistance in ascertaining the reason why wounds of the head should affect the liver in preference to other viscera.

Are there not some facts above detailed, which invite medical practitioners to new researches?

"Among the facts, which daily meet our eyes," Barthez says, "those ought be regarded as peculiarly useful, which are rare and singular, provided their *credibility* is sufficiently supported by facts which agree with those already known."

A little farther on, the same author adds; "If we succeed ceed in methodically uniting a great number of facts, we shall attain what Fontenelle suggested, that is to say, facts which existed separate and distinct from each other, will naturally return and unite again to form one body."

